# Measuring treatment response to advance precision medicine for multiple sclerosis

**DOI:** 10.1002/acn3.51471

**Published:** 2021-10-26

**Authors:** Peter A. Calabresi, Ludwig Kappos, Gavin Giovannoni, Tatiana Plavina, Irene Koulinska, Michael R. Edwards, Bernd Kieseier, Carl de Moor, Elias S. Sotirchos, Elizabeth Fisher, Richard A. Rudick, Alfred Sandrock

**Affiliations:** ^1^ Department of Neurology Johns Hopkins University School of Medicine Baltimore Maryland USA; ^2^ Neurologic Clinic and Policlinic Departments of Medicine, Clinical Research, Biomedicine and Biomedical Engineering University Hospital and University of Basel Basel Switzerland; ^3^ Blizzard Institute, Barts and The London School of Medicine and Dentistry Queen Mary University of London London UK; ^4^ Biogen Cambridge Massachusetts USA; ^5^ Department of Neurology Heinrich Heine Universitat Dusseldorf Germany

## Abstract

**Objective:**

To assess the independent contributions of clinical measures (relapses, Expanded Disability Status Scale [EDSS] scores, and neuroperformance measures) and nonclinical measures (new brain magnetic resonance imaging [MRI] activity and serum neurofilament light chain [sNfL] levels) for distinguishing natalizumab‐treated from placebo‐treated patients.

**Methods:**

We conducted post hoc analyses using data from the AFFIRM trial of natalizumab for multiple sclerosis. We used multivariable regression analyses with predictors (EDSS progression, no relapse, new or enlarging MRI activity, brain atrophy, sNfL levels, and neuroperformance worsening) to identify measures that independently discriminated between treatment groups.

**Results:**

The multivariable model that best distinguished natalizumab from placebo was no new or enlarging T2 or gadolinium‐enhancing activity on MRI (odds ratio; 95% confidence interval: 7.2; 4.7–10.9), year 2 sNfL levels <97.5th percentile (4.1; 2.6–6.2), and no relapses in years 0–2 (2.1; 1.5–3.0). The next best‐fitting model was a two‐component model that included no MRI activity and sNfL levels <97.5th percentile at year 2. There was little difference between the three‐ and two‐component models.

**Interpretation:**

Nonclinical measures (new MRI activity and sNfL levels) discriminate between treatment and placebo groups similarly to or better than clinical outcomes composites and have implications for patient monitoring.

## Introduction

Multiple sclerosis (MS) is the most common autoimmune disease of the central nervous system. During an initial relapsing‐remitting phase of the disease, episodic brain, and spinal cord inflammation typically manifest either as clinical relapses or as subclinical activity revealed by new or enlarging T2 hyperintense and gadolinium‐enhancing (Gd^+^) lesions on magnetic resonance imaging (MRI).^1^, ^2^ Gradually, the disease often evolves to a phase of worsening neurological and neuropsychological disability with fewer relapses and focal activity on MRI, and ongoing neurodegeneration manifested by brain atrophy.^3^, ^4^, ^5^ Optimal disease management requires close monitoring of disease activity and appropriate disease‐modifying therapy (DMT), with the goal of preserving brain tissue and cognitive and physical function. Recently, measurement of a structural axonal protein, neurofilament, in serum or plasma has shown promise as a marker of neuroaxonal injury and a measure of treatment response.^6^, ^7^

Clinical trials require the designation of a primary outcome measure on which treatment efficacy can be judged. The most common clinical trial outcomes include the frequency of clinical relapses and worsening on the Kurtzke Expanded Disability Status Scale (EDSS).^4^ Relapse rate and EDSS are universal measures in clinical trials for relapsing forms of MS and many experts believe that MS outcome assessment in clinical practice should include multiple measures that more completely reflect the complex disease process and its manifestations.^8^

A combination of traditional clinical trial measures (relapses, EDSS, and MRI lesions) is the basis for a composite outcome measure––no evidence of disease activity (NEDA)––first reported in post hoc analyses of the Natalizumab Safety and Efficacy in Relapsing‐Remitting Multiple Sclerosis (AFFIRM) study.^9^, ^10^ As initially reported, NEDA consisted of three variables (now referred to as NEDA‐3): (1) the absence of clinical relapse; (2) no disability progression measured using the EDSS sustained for ≥3 months; and (3) no new or enlarging T2 or Gd^+^ lesions.^9^ Recently, a fourth variable, annualized whole brain volume loss (BVL), has been introduced (referred to as NEDA‐4).^10^, ^11^ The inclusion of cerebrospinal fluid neurofilament light chain (NfL) as a fluid biomarker of neurodegeneration has been proposed as NEDA‐5.^10^, ^12^ The association of NEDA with long‐term progression is not yet established.^12^ Moreover, although individual NEDA components may be differentially associated with long‐term MS outcomes, each has equal weight in the current NEDA paradigm.^10^ There are also concerns that routine incorporation of NEDA into clinical practice is not feasible given that EDSS is not universally used by neurologists,^13^, ^14^ and because MRI lesion assessments can be unreliable because of a lack of standardized MRI acquisition and reporting.^15^ Given these limitations, uncertainty remains regarding whether the current versions of NEDA offer a practical and optimally informative treatment target for MS clinical practice.

Over the past 20 years, numerous DMTs for MS have been introduced to the market, leading to an increased need for outcome measures that can be applied at the individual patient level to move the field in the direction of precision medicine and, eventually, personalized medicine. For this purpose, candidate outcome measures should reflect its clinical impact; should be quantitative and reproducible; and should be available across practice settings, geographies, and cultures. Ideally, a treatment response tool should derive from the unbiased assessment of candidate variables, rather than the incorporation of primary and secondary clinical trial outcomes into a composite measure. To explore this concept, and to generate preliminary data that can be further tested in real‐world populations, we systematically assessed which measures collected during the AFFIRM trial of natalizumab^16^ best distinguished patients administered placebo or natalizumab.

## Methods

AFFIRM was a large, 2‐year, phase 3, randomized, placebo‐controlled study to evaluate natalizumab versus placebo in adults with relapsing MS (NCT00027300).^16^ During AFFIRM, data on relapses were recorded when they occurred, EDSS was scored at 12‐week intervals, and MRI scans were obtained at baseline, week 52, and week 104.^16^ Neuroperformance measures (Paced Auditory Serial Addition Test [PASAT], 9‐Hole Peg Test [9HPT], and Timed 25‐Foot Walk [T25FW]) were evaluated at 12‐week intervals.^17^ In addition, as part of retrospective analyses, serum neurofilament light chain (sNfL) levels were measured in biobanked serum samples collected at baseline and months 3, 6, 9, 12, 18, and 24, and then frozen at −70°C or −80°C, depending on the freezer used.

Endpoints were defined as per the AFFIRM study.^16^ Confirmed EDSS progression was defined as ≥1‐point increase from baseline EDSS score of ≥1.0 or a ≥1.5‐point increase from a baseline EDSS score of 0. Worsening required confirmation after 12 weeks.^16^ A relapse was defined as new or recurrent neurologic symptoms that persisted for ≥24 h, not related to a concurrent fever or infection, accompanied by new neurologic findings.^16^ Brain MRI (proton‐density‐weighted, T2‐weighted, and pre‐ and post‐gadolinium T1‐weighted image) using axial slices of 3‐mm thickness were acquired.^16^ Lesions were quantified by the central MRI reading center (University College London, London, UK) and brain volume was assessed using brain parenchymal fraction (BPF) at a separate MRI analysis center (Cleveland Clinic, Cleveland, OH, USA). sNfL was measured using the Simoa NF‐light™ Advantage Kit (Quanterix, Billerica, MA, USA).^18^ Worsening on PASAT, 9HPT, or T25FW was defined as worsening of ≥20% from baseline score sustained for ≥12 weeks.^19^ BVL was calculated as annualized BPF percentage change from year 1 to year 2. BVL was classified as low versus high using a threshold of less than −0.2%, based on the median BPF percentage change in the group treated with natalizumab in year 2.

**Figure 1:**
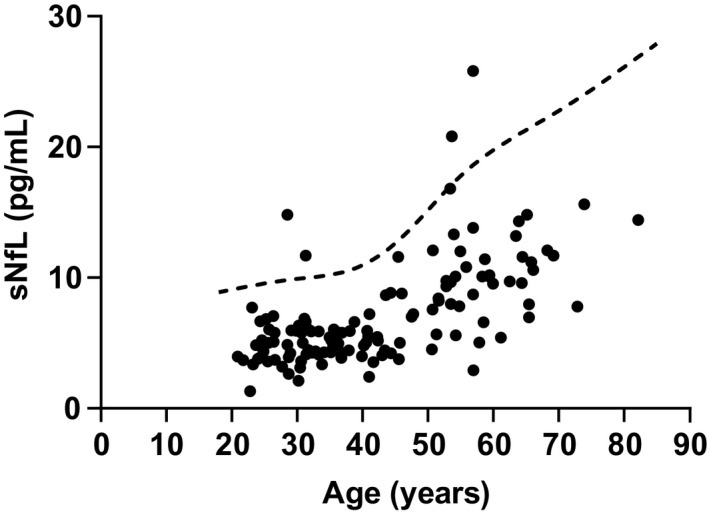
Serum neurofilament light chain (sNfL) levels from individual healthy controls (*N* = 130). Dashed line represents the age‐adjusted 97.5th percentile.

We evaluated sNfL data from 130 healthy controls recruited at Johns Hopkins University among hospital staff and nonconsanguineous family members of patients to define the age‐normative 97.5th percentile using the generalized additive model for location, scale, and shape (GAMLSS model; Fig. 1).^20^ For the data from participants in AFFIRM, sNfL levels were classified as normal (<97.5th percentile) or elevated (>97.5th percentile) based on these age‐normative data.

### Standard protocol approvals, registrations, and patient consents

AFFIRM study participants provided consent to participate in writing and to provide serum samples for possible use in future MS research.^16^ Participating sites approved the study protocol and the study was conducted according to the International Council for Harmonization Guideline on Good Clinical Practice and the Declaration of Helsinki. The healthy controls who provided samples for sNfL analysis provided written informed consent, and the protocol was approved by the institutional review board of Johns Hopkins University.

### Analyses

Change from baseline to year 2 was assessed for all measures except BVL, which was assessed for year 1–2 to avoid the confounding effect of pseudoatrophy in the first year of natalizumab treatment.^21^ sNfL was assessed only cross‐sectionally at the end of year 1 and year 2.

Comparisons between treatment groups for each measure included odds ratios (ORs) and area under the receiver‐operating characteristic curve (AUC), with treatment group as the dependent variable. Multivariable logistic regression analyses with eight predictors (no EDSS progression [3‐month confirmed], no relapse [0–2 years], no MRI activity, defined as no new or enlarging T2 or Gd^+^ lesions [0–2 years], annualized BVL less than –0.2% [year 1–2], sNfL <97.5th percentile at year 2 [metric based on largest sample size], or <20% confirmed worsening in PASAT, 9HPT, and T25FW scores) were used to evaluate which measures independently discriminated between treatment groups. Tenfold cross‐validation was used to partition the original sample into a training set to train the model and a test set to evaluate it.

Binary composites of individual measures from the best‐fitting models were compared with the composites NEDA‐3 and NEDA‐4. Binary composites were created in which a patient was assigned a “1” if they achieved the thresholds for all composite variables, and a “0” otherwise.

## Results

Baseline characteristics for all 942 participants in AFFIRM have previously been published.^16^ Baseline characteristics for the 792 (84%) AFFIRM participants who had sNfL measurements are shown in Table 1 (natalizumab [*n* = 537], placebo [*n* = 255]). Baseline characteristics shown in Table 1 are typical for a relapsing MS population.

**Table 1:** Baseline characteristics of patients for the MS disease control analysis.

Characteristic	Disease control population (*N* = 792)^1^
Mean (SD) age, years	36.2 (8.2)
Sex, *n* (%)	
Male	244 (31)
Female	548 (69)
Race, *n* (%)	
White	761 (96)
Other	31 (4)
Median disease duration, years	6
Number of relapses in prior year, *n* (%)	
0	9 (1)
1	462 (58)
2	260 (33)
≥3	61 (8)
EDSS score, *n* (%)	
0	44 (6)
1.0–1.5	240 (30)
2.0–2.5	261 (33)
3.0–3.5	162 (20)
4.0–4.5	65 (8)
5.0	18 (2)
≥5.0	2 (<1)
Mean (SD) BPF^2^	0.824 (0.022)
Mean (SD) T2 lesion volume, mm^3^	15,293.8 (16,559.2)
Patients with Gd^+^ lesions, *n* (%)	385 (48.6)
Mean (SD) sNfL, pg/mL	16.7 (21.1)

BPF, brain parenchymal fraction; EDSS, Expanded Disability Status Scale; Gd^+^, gadolinium‐enhancing; MS, multiple sclerosis; SD, standard deviation; sNfL, serum neurofilament light chain.

^1^ Natalizumab, *n* = 537; placebo, *n* = 255.

^2^ Ratio of BPF to total volume within the brain surface contour.

Table 2 shows the differences between treatment groups for the potential outcome variables (EDSS, T25FW, 9HPT, PASAT, number of relapses, MRI activity) collected during AFFIRM, and one variable (sNfL) measured in stored serum samples that were obtained during the trial. Univariate analyses showed that the three measures with the strongest association with natalizumab treatment assignment (OR; AUC; *p*‐value vs. placebo) were: (1) MRI activity (9.3; 0.73; *p* < 0.0001), (2) sNfL (<97.5th percentile at year 2: 6.1; 0.65; *p* < 0.0001; <97.5th percentile at year 2 and year 1: 4.5; 0.66; *p* < 0.0001; mean sNfL [year 2 and year 1] <97.5th percentile: 5.1; 0.64; *p* < 0.0001), and (3) the number of relapses during the 2‐year trial (1–2 years: 3.5; 0.62; *p* < 0.0001; 0–2 years: 2.7; 0.62; *p* < 0.0001). Measures of disease progression that had lower or no association with treatment were EDSS progression (1.7; 0.54; *p* = 0.0044), T25FW (1.5; 0.53; *p* = 0.047), rate of brain atrophy (91.3; 0.53; *p* = 0.14), and 9HPT (1.2; 0.51; *p* = 0.47).

**Table 2:** Univariate analysis: treatment group differences for individual measures of MS disease control.

Measure of disease control	Proportion of patients meeting the disease control measure, %		Measures of strength association			
	Natalizumab (*n* = 537)	Placebo (*n* = 255)	OR	*p*‐value	Difference	AUC
MRI activity^1^	59.4	13.6	9.3	<0.0001	45.8	0.73
sNfL <97.5th percentile at year 2	90.3	60.4	6.1	<0.0001	29.9	0.65
Mean (sNfL year 2 and sNfL year 1) <97.5th percentile	88.8	61.0	5.1	<0.0001	27.8	0.64
sNfL <97.5th percentile at year 2 and year 1	80.7	48.1	4.5	<0.0001	32.6	0.66
No relapse (1–2 years)	83.2	58.4	3.5	<0.0001	24.8	0.62
No relapse (0–2 years)	63.7	39.6	2.7	<0.0001	24.1	0.62
No EDSS progression	82.5	73.7	1.7	0.0044	8.8	0.54
T25FW (<20% worsening)	82.8	76.8	1.5	0.047	6.0	0.53
Annualized BVL <0.2% (1–2 years)	45.4	39.6	1.3	0.14	5.8	0.53
9HPT (<20% worsening)	88.4	86.6	1.2	0.47	1.8	0.51
PASAT (<20% worsening)	97.4	97.3	1.1	0.91	0.1	0.50
Annualized BVL <0.2% (0–2 years)	32.4	35.5	0.9	0.40	−3.1	0.52

9HPT, 9‐Hole Peg Test; AUC, area under the receiver‐operating characteristic curve; BVL, brain volume loss; EDSS, Expanded Disability Status Scale; Gd^+^, gadolinium‐enhancing; MRI, magnetic resonance imaging; MS, multiple sclerosis; OR, odds ratio; PASAT, Paced Auditory Serial Addition Test; sNfL, serum neurofilament light chain; T25FW, Timed 25‐Foot Walk.

97.5th percentile derived from Johns Hopkins University normative healthy control data set.

^1^ No new or enlarging T2 or Gd^+^ lesions.

Table 3 shows results from a multivariable model. When all candidate measures were included, only three remained significant: MRI activity, sNfL, and number of relapses.

**Table 3:** Multivariable logistic regression model: Association of measures of natalizumab treatment effect versus placebo.

Variable	OR	95% CI	*p*‐value
MRI activity^1^	6.9	4.5–10.6	<0.0001
sNfL<97.5th percentile at year 2^2^	3.8	2.4–5.9	<0.0001
No relapse (0–2 years)	1.8	1.2–2.6	0.0026
No EDSS progression	1.5	0.9–2.4	0.1043
T25FW (<20% worsening)	1.0	0.6–1.6	0.9663
PASAT (<20% worsening)	0.9	0.3–2.8	0.8068
Annualized BVL <0.2% (1–2 years)	0.9	0.6–1.4	0.7350
9HPT (<20% worsening)	0.8	0.5–1.5	0.5219

9HPT, 9‐Hole Peg Test; BVL, brain volume loss; CI, confidence interval; EDSS, Expanded Disability Status Scale; Gd^+^, gadolinium‐enhancing; MRI, magnetic resonance imaging; OR, odds ratio; PASAT, Paced Auditory Serial Addition Test; sNfL, serum neurofilament light chain; T25FW, Timed 25‐Foot Walk.

97.5th percentile derived from Johns Hopkins University normative healthy control data set.

^1^ No new or enlarging T2 or Gd^+^ lesions.

^2^ sNfL metric based on largest sample size.

Table 4 shows the best‐fitting multivariable logistic regression model. Included in the model (OR; 95% CI) were no MRI activity (7.2; 4.7–10.9), sNfL (<97.5th percentile at year 2, 4.1; 2.6–6.2), and no relapses at years 0–2 (2.1; 1.5–3.0; *p* < 0.0001). The next best‐fitting model included no MRI activity (7.9; 5.2–11.9) and sNfL (<97.5th percentile at year 2, 3.3; 2.4–4.7).

**Table 4:** Best‐fitting three‐variable multivariable logistic model predicting natalizumab versus placebo.

Variable	OR	95% CI	*P*‐value
MRI activity^1^	7.2	4.7–10.9	<0.0001
sNfL <97.5th percentile at year 2	4.1	2.6–6.2	<0.0001
No relapse (0–2 years)	2.1	1.5–3.0	<0.0001

CI, confidence interval; Gd^+^, gadolinium‐enhancing; MRI, magnetic resonance imaging; OR, odds ratio; sNfL, serum neurofilament light chain.

97.5th percentile derived from Johns Hopkins University normative healthy control data set.

^1^ No new or enlarging T2 or Gd^+^ lesions.

**Figure 2:**
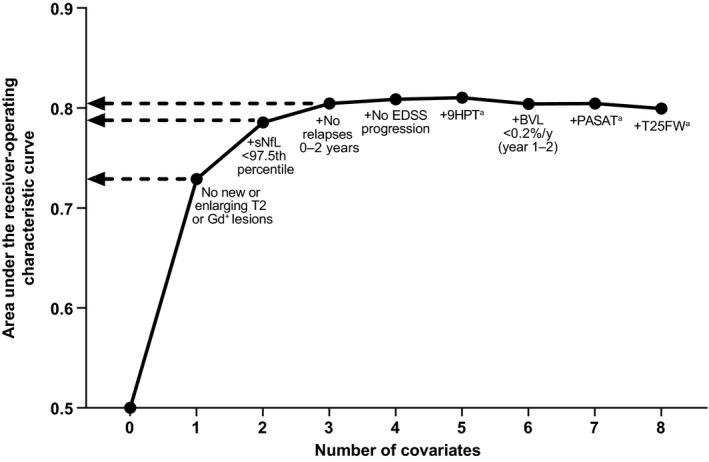
Area under the receiver‐operating characteristic curve × number of covariates from the multivariable model predicting natalizumab versus placebo. 9HPT, 9‐Hole Peg Test; BVL, brain volume loss; EDSS, Expanded Disability Status Scale; Gd^+^, gadolinium‐enhancing; PASAT, Paced Auditory Serial Addition Test; sNfL, serum neurofilament light chain; T25FW, Timed 25‐Foot Walk. ^a^<20% worsening.

To show the association between the number of covariates in the logistic regression models and the model AUC, a backward stepwise elimination was implemented beginning with the full model containing all eight statistically significant predictors from the univariate associations. Fig. 2 shows the contribution of each predictor to the model AUC. The improvement in AUC plateaued after the best three variables in the model (no MRI activity, sNfL <97.5th percentile, and relapses). Two of these variables (MRI activity and sNfL) had an AUC of 78.9. The addition of relapses to the model increased the AUC to 80.8 and the addition of the other variables marginally improved the AUC to ˜81, where it also peaked. This indicates that nearly all of the ability to distinguish between the groups was derived from two nonclinical disease activity measures.

In order to compare with NEDA, a binary variable was created in which a patient either met all of the criteria represented by the dichotomous variables in the model or failed to meet at least one variable. Analyses of treatment group differences of binary measures are shown in Table 5. For inclusion in the two‐component binary composite of disease control, patients were required to have both (1) no MRI activity and (2) sNfL at year 2 of <97.5th percentile. For the three‐component binary composite of disease control, a patient was required to have (1) no MRI activity, (2) sNfL at year 2 of <97.5th percentile, and (3) no relapses from year 0 to year 2. The two‐component binary composite had an AUC of 71.3 and the three‐component composite had an AUC of 64.9. Both the two‐ and three‐component disease control models achieved similar treatment group discrimination as the NEDA three‐ and four‐component binary composites.

**Table 5:** Treatment group discrimination achieved by different models of composite MS disease control measures.

Binary composite	Proportion of patients meeting the criteria, %		Measures of group differences			
	Natalizumab (*n* = 537)	Placebo (*n* = 255)	OR	*p*‐value	Difference	AUC
NEDA three‐component binary composite: MRI activity, no relapses, and no EDSS progression	34.7	7.2	6.9	<0.0001	27.5	63.8
NEDA four‐component binary composite: MRI activity, no relapses, no EDSS progression, and BVL	16.9	3.4	5.8	<0.0001	13.5	56.7
Disease control two‐component binary composite: MRI activity and sNfL <97.5th percentile at year 2	55.0	12.4	8.6	<0.0001	32.6	71.3
Disease control three‐component binary composite: no relapses, MRI activity, and sNfL <97.5th percentile at year 2	36.6	6.8	7.9	<0.0001	29.8	64.9

AUC, area under the receiver‐operating characteristic curve; BVL, brain volume loss; EDSS, Expanded Disability Status Scale; MRI, magnetic resonance imaging; MS, multiple sclerosis; NEDA, no evidence of disease activity; OR, odds ratio; sNfL, serum neurofilament light chain.

97.5th percentile derived from Johns Hopkins University normative healthy control data set.

In addition to the analytic derivation of the composite measures, a sensitivity analysis was conducted in which all 255 of the possible combinations of the eight outcome measures were evaluated. Each of the possible composites were evaluated using cross‐validation, and the best‐performing measures were again found to be the three‐ and two‐component composites.

## Discussion

Using data from the 2‐year AFFIRM study and a data analytic approach, we showed that MRI activity (new or enlarging T2 or Gd^+^ lesions), sNfL levels, and relapses had the strongest association with natalizumab treatment. Furthermore, a majority of the probability of distinguishing natalizumab treatment from placebo was explained by MRI activity and sNfL alone; relapses added only slightly to this probability. Short‐term measures of disease progression as assessed by concurrent changes in EDSS progression, 9HPT, T25FW, PASAT, and brain atrophy provided minimal additional value in discriminating between patients on natalizumab versus placebo. This suggests that measures commonly associated with disease progression may not closely reflect treatment with a potent anti‐inflammatory DMT, at least during the first 2 years of treatment. These results are consistent with current concepts about MS pathogenesis, which suggest that inflammatory activity leads to cumulative tissue destruction downstream.

We also report that a two‐component composite consisting of MRI lesion activity and sNfL, or a three‐component composite also including relapses, discriminated between placebo and natalizumab treatment as well as or better than NEDA‐3 and NEDA‐4. This suggests that measuring EDSS or brain atrophy is not as sensitive as MRI lesion activity and sNfL for monitoring the effect of natalizumab on disease activity. As demonstrated in the AFFIRM trial, natalizumab treatment was associated with a significantly lower likelihood of confirmed EDSS worsening and lower brain atrophy in year 2 of the trial. The current study results are fully consistent with findings from the clinical trial, further suggesting that the primary effect of natalizumab is on inflammation, and that the demonstrated effects on disability progression and brain atrophy are secondary benefits resulting from the anti‐inflammatory effects of natalizumab.

Our results suggest a role for sNfL levels as a biomarker of neuroaxonal damage and disease activity in the early assessment of natalizumab treatment. Neurofilaments are structural scaffolding proteins of the neurons and are released in response to neuroaxonal damage.^22^ Elevated levels of NfL have been detected in the cerebrospinal fluid and serum of patients with MS, and it has been suggested as a prognostic marker for MS.^22^ In patients with MS, higher sNfL levels correlated with clinical and imaging measures of disease severity, including brain and spinal cord volume loss.^22^, ^23^, ^24^, ^25^ sNfL levels decrease in patients with MS treated with DMTs.^22^, ^23^, ^24^ Changes in sNfL levels can be easily measured in blood samples with high reliability and sensitivity using recently developed bioassays.^24^, ^26^, ^27^ Up‐to‐date evidence shows that higher sNfL levels may also be an indicator of suboptimal drug response^28^ and disease activity when routine clinical and MRI assessment produce false negatives.^29^ The integration of sNfL as a blood‐based biomarker in MS clinical practice will be dependent on the technical and clinical validation of sNfL as a diagnostic test, improved understanding of confounding variables such as comorbid illnesses and body mass index, and, finally, the establishment of normal age‐related reference values.^30^ Thereafter, a simple blood test to measure sNfL levels could complement MRI in monitoring the effectiveness of natalizumab and possibly other anti‐inflammatory DMTs.

Brain atrophy can be seen in the earliest stages of MS and predicts future cognitive and physical disability.^31^, ^32^ BVL values depend on the methodology used to generate them. The image analysis techniques, and to a lesser extent the image acquisitions, have a significant impact on volumetric measurements. The optimal threshold for BVL is not yet clear and it is possible that results may change with the use of an alternative threshold.

BPF, the ratio of brain parenchymal volume to total volume within the brain surface contour, has been previously used to quantify brain atrophy in patients with MS.^32^ Not only are changes in BPF in patients with early MS predictive of future cognitive and physical impairment, but they can also serve as an indirect measure of neurodegeneration in MS.^31^, ^33^ In AFFIRM, there was a significant reduction in brain atrophy in the natalizumab group versus placebo in the second year of the trial.^34^ The finding that BPF changes were not highly predictive of natalizumab treatment in the present modeling approach 2‐year analysis may be due to the fact that brain atrophy occurs as a secondary, downstream consequence of inflammatory activity,^22^ and, therefore, it is not surprising that BPF change did not add much to the model beyond new T2 lesions, sNfL, and relapses.

Notably, relapses improved AUC by only 1.8 when added to the two‐variable model containing MRI activity and sNfL data. In clinical practice, relapses remain difficult to clearly define and are subjective—one patient’s interpretation of their symptoms and their own personal threshold for reporting may differ widely from the next patient’s.^35^ Given the variable time allotted for patient encounters, imperfect recall by patients, and misclassification of functional symptoms as a relapse, it may be challenging to standardize both the definition and detection of relapse in the clinical setting.

There are a number of limitations of this study, including that it was conducted over 2 years and reflects short‐term variables such as inflammatory markers. The study findings should be assessed over longer time frames, after which other measures, such as BPF and cognition, may add increased relevance and stronger contributions to the model. In addition, the data presented are from post hoc analyses of a clinical trial and, although informative, should be confirmed in a real‐world setting across a broader range of MS clinical subtypes. In AFFIRM, 6% of the patients treated with natalizumab developed persistent antibodies to natalizumab^16^; our sampling did not exclude these patients. Furthermore, this study addresses the value of different prognostic factors in differentiating natalizumab from placebo and cannot automatically be generalized to other treatments with different effect sizes or modes of action. Ongoing studies will add to our understanding of whether patients meeting the criteria defined by sNfL and MRI measurements (new and enlarging T2 and Gd^+^ lesions) alone will have better long‐term outcomes than predicted using the original NEDA measurement.

In conclusion, the combination of sNfL threshold and MRI activity yielded similar results to NEDA in predicting natalizumab treatment, and may prove to be more practical for individual monitoring of therapeutic response in clinical practice. New and enlarging T2 lesions and sNfL could potentially enter practice as standardized metrics, and could provide tools to monitor individual responses to anti‐inflammatory therapy in the early stages of MS.

## Conflict of Interest

PAC: received consulting fees from Biogen, Disarm Therapeutics, and NervGen Pharma; grants from Annexon, Biogen, and Genentech. LK: University Hospital Basel received in the past 3 years and used exclusively for research support: steering committee/advisory board/consultancy fees from Actelion, Alkermes, Almirall, Bayer, Biogen, Celgene/Receptos, df‐mp, Excemed, GeNeuro, Japan Tobacco, Merck, Minoryx, Mitsubishi Pharma, Novartis, Roche, Sanofi, Sanofi‐Genzyme, Santhera, Teva, and Vianex; royalties for Neurostatus‐UHB Products and Services. GG: honoraria from AbbVie, Actelion, Atara Biotherapeutics, Bayer, Biogen, Canbex, FivePrime, GlaxoSmithKline, GW Pharma, Merck Serono, Novartis, Protein Discovery Laboratories, Roche, Sanofi‐Genzyme, Synthon, Teva Neuroscience, UCB Pharma, and Vertex Pharmaceuticals; compensation from Elsevier as co‐chief editor of *Multiple Sclerosis and Related Disorders*; research grant support from Biogen, Ironwood, Merck Serono, Merz, and Novartis. TP, IK, BK, RAR, and MRE: Biogen employees at the time of the study, with stock/stock options in Biogen. ESS: advisory boards for Alexion, Genentech, and Viela Bio; speaker honoraria from Biogen and Viela Bio. CdM, EF, and AS: employees of and hold stock in Biogen.

## Author Contributions

Analyses were conducted by CdM and ESS. All authors participated in the interpretation of the data, contributed to reviews of the manuscript, and approved the final version for submission.
